# Heart Rate during Conflicts Predicts Post-Conflict Stress-Related Behavior in Greylag Geese

**DOI:** 10.1371/journal.pone.0015751

**Published:** 2010-12-20

**Authors:** Claudia A. F. Wascher, Orlaith N. Fraser, Kurt Kotrschal

**Affiliations:** 1 Konrad Lorenz Forschungsstelle, Grünau im Almtal and Department of Behavioural Biology, University of Vienna, Vienna, Austria; 2 Department of Cognitive Biology, University of Vienna, Vienna, Austria; University of Lethbridge, Canada

## Abstract

**Background:**

Social stressors are known to be among the most potent stressors in group-living animals. This is not only manifested in individual physiology (heart rate, glucocorticoids), but also in how individuals behave directly after a conflict. Certain ‘stress-related behaviors’ such as autopreening, body shaking, scratching and vigilance have been suggested to indicate an individual's emotional state. Such behaviors may also alleviate stress, but the behavioral context and physiological basis of those behaviors is still poorly understood.

**Methodology/Principal Findings:**

We recorded beat-to-beat heart rates (HR) of 22 greylag geese in response to agonistic encounters using fully implanted sensor-transmitter packages. Additionally, for 143 major events we analyzed the behavior shown by our focal animals in the first two minutes after an interaction. Our results show that the HR during encounters and characteristics of the interaction predicted the frequency and duration of behaviors shown after a conflict.

**Conclusions/Significance:**

To our knowledge this is the first study to quantify the physiological and behavioral responses to single agonistic encounters and to link this to post conflict behavior. Our results demonstrate that ‘stress-related behaviors’ are flexibly modulated by the characteristics of the preceding aggressive interaction and reflect the individual's emotional strain, which is linked to autonomic arousal. We found no support for the stress-alleviating hypothesis, but we propose that stress-related behaviors may play a role in communication with other group members, particularly with pair-partners.

## Introduction

In group-living animals, including greylag geese, agonistic encounters and other social interactions are common and have been shown to modulate an individual’s short-term stress response [1], [2] by activating both the hypothalamic-pituitary-adrenal (HPA) axis and the sympathico-adrenergic system. Furthermore, stressful events may also affect an individual’s behavior. In particular, certain ‘self-directed behaviors’ (SDBs, e.g. scratching, preening), termed ‘displacement behaviors’ in the classical ethological literature [3], have been suggested to function as indicators of individual emotional tension and may be related to autonomic arousal [4]. The occurrence of such behaviors may provide information about an individual's evaluation of a certain event [5], [6], [7].

In humans, ‘displacement behaviors’ have been shown to be causally linked to autonomic arousal and they provide more reliable information about an individual's emotional state than a verbal statement or facial expression [8]. In non-human animals, SDBs have been used to assess individual stress levels in response to a variety of potential stressors [9]. Acute crowding in European starlings (*Sturnus vulgaris*) has been shown to increase both physiological (heart rate and corticosterone) and behavioral (upright posture, headfeather expansion, pecking frequency) indicators of stress [10], [11], whereas different other acute stressors (e.g. strobe light, music, human disturbance) lead to an increased physiological stress response but caused a decrease in preening, beak wiping and feeding [12]. This suggests a direct link between physiological and behavioral stress responses, although behavioral responses could be bidirectional, causing an increase or a decrease in the physiological stress response to stressful events. The connection between the physiological stress response and single social events (e.g. agonistic encounters) is still unclear in non-human animals, probably because of the difficulty in obtaining data such as, heart rate measurements in free-living animals. In addition, fecal glucocorticoid metabolite measurements do not provide a sufficient temporal resolution to correlate with behavioral responses to single events. Nevertheless, the link between SDBs and emotional tension has been reinforced by evidence that self-scratching in primates increases on administration of anxiogenic (anxiety-inducing) drugs and decreases following administration of anxiolytic (anxiety-reducing) drugs [4], [13]. Moreover, these behaviors have been shown to vary according to the activity of the arginine vasotocin-system, at least in some male birds [14].

On the proximal level, such SDBs have been suggested to have a stress-alleviating function to keep an animal within optimal physiological and psychological limits [15], whereas on the ultimate level, such behaviors have been linked with social communication [4]. In particular, post-conflict affiliative interactions (such as reconciliation, i.e. post-conflict affiliation between former opponents, and consolation, i.e. an affiliative interaction directed from a third party towards the recipient of aggression [16] have been suggested to alleviate post-conflict stress. Support for this assumption is provided by the fact that SDBs remain elevated following conflicts after which post-conflict affiliation does not occur, but have been shown to reduce to baseline levels after reconciliation [17], [18], [19] or consolation [20]. The stress-alleviating effect of reconciliation has been additionally demonstrated using physiological measures (levels of salivary cortisol) in humans [21], but not in non-human animals.

In the present study, we investigated the contingencies between heart rates (HR) during an agonistic encounter and post-conflict behavior in greylag geese (*Anser anser*). To our knowledge, this the first study in non-human animals to investigate the correlation between stress physiology and behavior shown after the conflict in a non-human animal. Greylag geese are characterized by a relatively complex social system [22]. Individuals form long-term valuable relationships with their partner and/or offspring, showing spatial proximity and supporting each other in aggressive encounters [23], [24], [25], [26]. Geese form a dominance hierarchy, agonistic encounters are quite common and are known to strongly modulate HR [27]. The degree of HR modulation depends on the characteristics of the interaction (e.g. its intensity) as well as the identity of the opponent (e.g. sex, rank: 1). In the present study, we aimed to find out whether the degree of HR modulation during agonistic encounters and the time until HR reaches baseline levels after the conflict would predict the frequency of post-conflict behaviors (body shaking, autopreening, self-scratching, vigilance) and of post-conflict distance between partners. We expected the frequency and duration of certain behaviors (e.g. shaking, preening, vigilance) to vary in accordance with the degree of stress (as reflected in HR) elicited by a specific encounter. Longer and more intense interactions, likely to be accompanied by a stronger physiological response, were expected to elicit higher rates of SDBs.

## Methods

### Ethical Statement

The implantation of heart rate transmitters into the abdominal cavity of geese was conducted under a license issued by the Austrian Ministry of Science (GZ68.210/41-BrGT/2003). Implantations were conducted according to veterinarian state of the art techniques and all geese recovered within less than 24 hours. No other manipulations of the geese, which would have required additional licenses, were conducted.

### Study site

A non-migratory flock of greylag geese was introduced to the Almtal region of Upper Austria by Konrad Lorenz in 1973. The geese are unrestrained and roam the valley between the Konrad Lorenz research station and a lake approximately 10 km to the south, where they roost at night. At the time of data collection, the flock consisted of approximately 150 individuals, marked with colored leg bands for identification. The study population lives in an un-manipulated social environment and is free to choose with whom to interact, and whom to avoid [22]. Approx 30% of the geese in this flock are hand-raised. This, however, has been shown to have little impact on their social behavior [28].

The flock is supplemented with pellets and grain twice daily at 0800 and 1500 hours during the winter months and at 0800 and 1700 hours during the summer months. Both hand-raised and goose-raised flock members are habituated to the close presence of humans and they do not show avoidance, changes in agonistic behavior [29], elevated levels of corticosterone metabolites [30] or HR change when familiar humans approach to within 1 m [2].

### Transmitter technology and implantation technique

Twenty-five individuals were implanted with sensor-transmitter packages weighing approximately 60 g, which represents about 2% of their average body weight, with a battery lifetime of approximately 18 months, without external antennae or repeaters. General information about focal individuals (sex, pair bond status and baseline HR) is given in Table 1. Both HR and body core temperature were instantaneously transmitted on a beat by beat basis over distances up to 100 m and were stored as 2 min means in the implant over its lifetime [31]. The transmitter was calibrated to record and transmit in the range of 30 to over 500 beats per minute. An industrial tracking receiver module and a control board developed at the Research Institute of Wildlife Ecology was used to receive HR and body temperature data. Data were transferred to a commercial laptop computer via a serial interface (RS 232).

**Table 1:** General information about the focal individuals.

Focal	Sex	Pairing status	Mean partner distance (cm)	Index of involvement	Number of agonistic interactions		Mean MC HR^1^
					Aggressor	Target	
Armando	female	paired	83.82	0.01	1	0	140.46
Balu	female	paired	120	0.04	5	1	119.02
Blossom	male	single	-	0.02	1	1	113.47
Boston	male	single	-	0.1	6	5	119.63
Celine	female	paired	26.94	0.02	3	1	101.99
Corrie	male	paired	51.78	0.05	2	4	118.35
Edes	male	single	-	0.1	3	2	102.09
Halas	male	paired	130	0.01	2	0	106.57
Jacky	female	paired	29.67	0.01	1	0	82.76
Jana	female	paired	272.22	0.03	1	2	105.23
Jesaja	female	paired	138.76	0.06	2	4	125.74
Juniper	male	single	-	0.01	1	0	100.8
Keiko	male	paired	254.36	0.06	4	3	109.45
Little	male	single	-	0.03	1	3	105.02
Loki	male	paired	35.23	0.1	1	1	149.84
Pilvi	male	paired	100.68	0.09	16	8	98.71
Smoky	male	paired	105.53	0.02	0	2	86.02
Stella	male	paired	36.42	0.06	5	7	88.49
Terri	male	paired	142.52	0.09	4	3	119.81
Therese	male	paired	36.3	0.06	24	8	100.64
Tian	male	paired	135.59	0.03	0	1	106.64
Tristan	male	paired	37.97	0.03	2	2	129.03

1 Mean heart rate during the two minute matched control period recorded prior to the conflicts.

Implantations were conducted from 18 May to 14 September 2005 in five sessions of five individuals per session. Geese were caught opportunistically on the day before implantation or on the same day in the morning and kept together in a large outdoor aviary with access to a small pond and food or were isolated in transport boxes (depending on the time until surgery). The electronic package was implanted into the abdominal cavity by an experienced team of veterinarians in a properly equipped surgery room at the Cumberland Veterinary Clinic in Gmunden, the closest clinic to the study site. For details about the anesthesia and surgery, please see Wascher et al. [1].

After surgery, geese were housed individually in transport boxes overnight to recover from anesthesia. All 25 individuals were released into the flock the morning after implantation, after veterinary inspection. They returned to, and were accepted by, their social partners immediately after release. One female disappeared the night after release and was probably taken by a fox, the main natural predator of geese at the study site. The geese had fully recovered from the surgery after 2 to 7 days (flying as untreated animals, normal body temperatures and HRs). They could not be distinguished from non-implanted geese visually or in behavior (unpublished data). To exclude possible effects of the surgery on behavior or HR, we excluded from our analyses all data obtained within three weeks post surgery.

### Data collection

The study period lasted for 18 months between June 2005 and November 2006 during which the HR and behavior [32] of 22 focal individuals (16 males, 6 females) were recorded simultaneously by one of us (C.A.F.W.) at a maximal distance of 10 m from the focal individual. Individual observation sessions were recorded between 0830 and 1900 and the duration varied between 5 to 120 minutes, depending upon context (e.g. resting or feeding). We recorded all agonistic encounters in which a focal individual was involved, regardless of whether the focal attacked an opponent or was attacked by another flock member. For all interactions reported here, the attacker also won the interaction. We differentiated between four intensities of agonistic encounters (see Table 2 for definitions). For the present analysis we only included interactions that lasted at least 3 seconds (N = 143). We applied a slightly modified version of the standard ‘post-conflict (PC)- matched-control (MC)’ method commonly used to investigate post-conflict interactions [33], where each PC was a two-minute focal sample on one of the conflict participants, during which all behaviors exhibited by the focal individual (e.g. preening, shaking (body, tail, neck), feeding, vigilance) and the distance to the partner were recorded (Table 1). Similar data were collected during the two minutes preceding the conflict (‘matched control’ period) to provide baseline data. We choose two-minute PC and MC periods as HR responses most often occur within the first few seconds of the event. In 47.2% of all recorded interactions, HR reached baseline values usually within the first 10 seconds after aggression. Reaching baseline values took longer than one minute in only 14.4% of cases. The longest recorded HR increase was 95 seconds after the conflict. From the recordings collected during the entire study period (286h of data collection), we calculated an index of involvement (mean number of interactions per minute of observation) and mean inter-partner distance for each focal individual. Recordings were taken within a radius of 1.5 km around our research station.

**Table 2:** Description of variables used in the Linear Mixed Models and Generalized Linear Mixed Models.

Dependent variables	Autopreening	Occurrence of post-conflict autopreening (yes/no)
	Vigilance	Time spent (s) with head up in vigilant posture
	Shaking	Frequency of post-conflict shaking of the tail, neck or complete body
	Interpartner distance	Minimum distance between pair partners during post-conflict period
Predictor variables	Mean HR	Mean heart rate during conflict
	Maximum HR	Maximum heart rate during conflict
	HR increase	Difference between baseline heart rate before conflict and maximum heart rate
	Time until baseline	Time until heart rate reaches baseline levels again
	Sex	Male or female
	Intensity	1 = threat posture without locomotion; 2 = walking approach in threat posture; 3 = running or flying towards opponent; 4 = contact aggression (biting or wing beating
	Duration	Duration of the interaction in seconds
	Attacking versus being attacked	Focal individual initiating or being involved in the interaction
	Involvement	Mean number of interactions per observed minute, over the entire 18 months observation period
	Mean partner distance	Estimation of mean partner distance during the entire observation time of 18 months
Random effects	Individual identity	Focal individual
	Baseline pre-conflict heart rate	Mean heart rate 120 seconds until 30 seconds before the conflict

### Analysis

Comparison of the frequency and duration of post-conflict stress-related behaviors (shaking, vigilance and preening) and mean partner post-conflict distances with pre-conflict matched control sequences were performed using Wilcoxon signed rank tests.

Linear mixed models (LMMs) were used to investigate the influence of conflict characteristics (conflict intensity, duration, attacking versus being attacked, mean HR, max HR, HR increase, time until baseline HR is reached again) and the individual characteristics (sex, baseline HR, mean partner distance, involvement in agonistic encounters) on rates of shaking and vigilance during the post-conflict period. The dependent variables were subjected to log transformations to ensure model residuals conformed to a normal distribution. As preening during the post-conflict period occurred rarely, it was converted into a binomial variable (preening/no preening) and entered as a dependent variable in a generalized linear mixed model (GLMM) with the same predictor variables as for the LMMs. A further GLMM was run with partner distance (approach/no approach) as a dependent variable. GLMMs were fit by the Laplace approximation. Subject and opponent identity were included as random factors in all models to control for between-subject variation and non-independence of data points. We used the Spearman's rank correlation to explore the correlations between the predictor variables. Any highly correlated variables (|r_s_|>0.9) were not included in the same model. For each dependent variable we ran models with all possible combinations of predictor variables (see Table 2 for all variables included). We selected the best model using Akaike's information criteria (AIC), which compares the adequacy of several models and identifies the model that best explains the variance of the dependent variable as that with the lowest AIC value. Where AIC values between models vary by less than 2, those models are not considered to be significantly different in the adequacy with which they explain any variance in the dependent variable. In such cases, the model with the fewest predictor variables was chosen as the most parsimonious model is always preferred [34], [35]. Only the effects of those variables present in the best model are presented here. All LMMs were conducted in SPSS v. 17 and GLMM were conducted in the lme4 package in R v. 2.10.1.

## Results

Shaking behavior occurred significantly more often in the post-conflict (PC) period compared to matched control (MC; mean±S.D.: MC: 0.72±1.16, PC: 2.05±3.71; Wilcoxon signed rank test: n = 22, Z = 2.588, p = 0.011). Neither the occurrence of the other behaviors (vigilance: n = 22, Z = −1.705, p = 0.088; mean±S.D.: MC: 9.39±5.31, PC: 10.65±4.95; preening: n = 22, Z = −0.719, p = 0.472; mean±S.D.: MC: 1.24±2.06, PC: 1.28±1.58) nor inter-partner distance (n = 21, Z = −1.111, p = 0.267; mean±S.D: MC: 64.88±37.08, PC: 62.75±33.77) varied between PCs and MCs.

**Figure 1:**
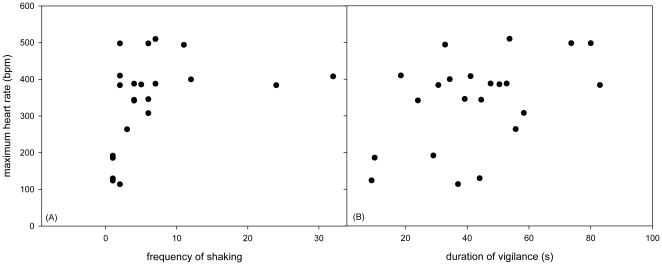
Correlation between maximum heart rate and mean individual frequency of post-conflict shaking (A) and duration of post-conflict vigilance (B).

**Figure 2:**
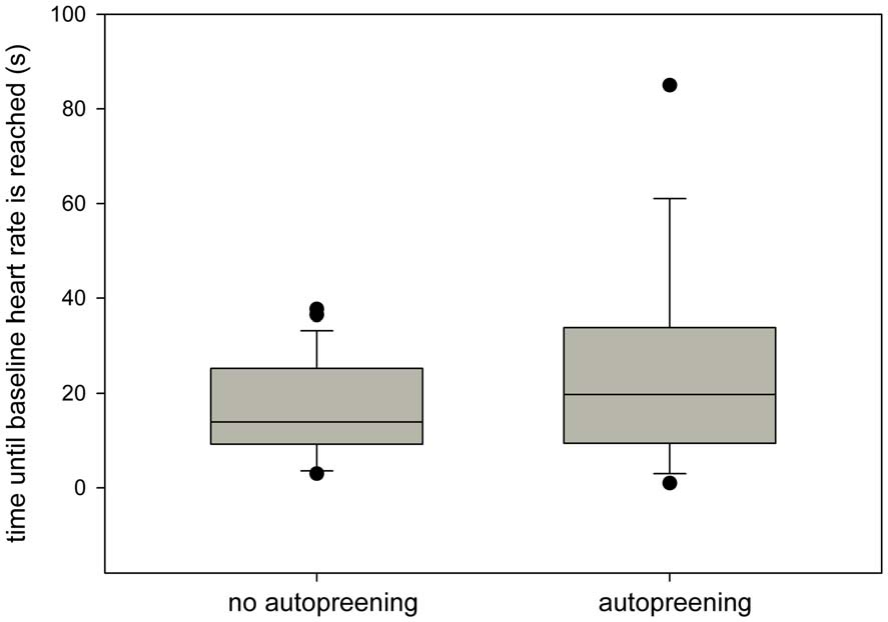
Median individual time until baseline heart rate is reached after an agonistic encounter when autopreening does and does not occur.

The frequency of shaking in the post-conflict period was positively influenced by the maximum HR (LMM: β = 0.0005, SE = 0.0002, t = 2.307, p = 0.023; Fig. 1A), by the intensity of the encounter (β = 0.105, SE = 0.05, t = 2.104, p = 0.037) and by the duration of the conflict (β = 0.006, SE = 0.003, t = 1.89, p = 0.061). The duration of vigilance behavior after a conflict increased with maximum HR (β = −0.132, SE = 0.059, t = −2.23, p = 0.027; Fig. 1B), with HR increase (β = 0.123, SE = 0.049, t = 2.509, p = 0.013), with the intensity of a conflict (β = 11.014, SE = 5.118, t = 2.152, p = 0.033) and with the involvement in agonistic encounters (β = −233.033, SE = 89.657, t = −2.599, p = 0.016). The occurrence of autopreening after a conflict was more likely when the focal individual was attacking compared to being attacked (GLMM: β = −1.004, SE = 0.478, z = −2.1, p = 0.035) and the time until HR reached baseline levels again was longer when autopreening occurred (β = 0.018, SE = 0.008, z = −2.101, p = 0.035; Fig. 2). Individuals who spent more time being vigilant during the post-conflict period were more likely to be closer to their partner than during the pre-conflict period (GLMM: β = 0.02, SE = 0.006, z = 3.044, p = 0.002).

## Discussion

For the first time in a non-human animal species, we relate individual heart rates (HR) shown during single agonistic encounters to the behavior shown after the conflict. We found a correlation between HR changes during or after a conflict and the occurrence of ‘stress-related behaviors’, such as body shaking, vigilance and autopreening. HR and the characteristics of an event such as conflict intensity and conflict duration reflect its relevance for the individual [1]. As such, our results support previous assumptions that SDBs are linked to autonomic arousal [4].

We can only speculate about the possible functions of the increase in SDBs after conflicts. However, our present results do not support a stress alleviating function of post-conflict SDBs in greylag geese, as HR did not decrease faster in post-conflict periods characterized by a higher rate of shaking, vigilance behavior or auto-preening. On the contrary, our results indicate that the occurrence of post-conflict autopreening actually prolonged the time it took for HR to reach baseline levels again. We cannot exclude the possibility that preening caused an increased HR due to subtle physical activity [36], but previous findings in greylag geese generally suggest that psychological factors play a more important role in modulating HR than physical activity [1], [37]. Also, we found preening to occur more likely after the focal individual was actively attacking than being attacked. This indicates that the emotional arousal is more pronounced in the attacker, than in the attacked, which may be explained by the level of motivation needed to initiate an attack and indicates that the internal tension is not entirely decomposed by the attack. The target individual however, sustained a lower level of arousal than the attacker before and during the attack and hence, ended after the attack with less internal tension than the attacker. These differences in PC behavior parallel differences in HR [27]. Given our results, however, we cannot distinguish between the physical and psychological factors that might account for the post-conflict HR decrease.

It seems likely that post-conflict stress alleviation is not mediated by post-conflict displacement behaviors but through some other mechanisms, such as through consolation from the pair partner. In the present study, however, the post-conflict inter-partner distance relative to their pre-conflict distance was not influenced by HR during the encounter or conflict characteristics (e.g. intensity, duration). Despite this, in 29% of encounters, conflict participants were closer to their partners after the conflict, although those encounters showing an increased post-conflict interpartner distance were not characterized by a higher HR response during the encounter, suggesting that partners were not more likely to approach after more stressful conflicts. Furthermore, as post-conflict HR did not predict interpartner distance, our data do not indicate that a target's post-conflict stress levels can be alleviated though close proximity to the partner. As it has been previously shown that HR and hormonal responses to stressors are regulated independently (12{Kralj-Fiser, 2010 #610}), SDBs could have a stress alleviating function through the hypothalamic–pituitary–adrenocortical system but not the sypathico-adrenergic system.

Another frequently discussed function for the occurrence of SDBs is social communication. In a range of animals, including greylag geese, an individual's behavior and physiology are not only affected by active involvement in an event but also just by witnessing an encounter between conspecifics [37], [38], [39]. Emotional contagion, a simple form of empathy, has been suggested as the mechanism behind this behavioral and physiological change [40]. A spontaneous impulse in reaction to distress signals of another individual (‘altruistic impulse’: [41]) may promote stress-alleviating mechanisms, e.g. consolation, from bystanders towards a target of aggression [42], or it may give uninvolved individuals the chance to prepare for action [43]. Distress contagion is described in a predator avoidance context [44], [45], [46], but it might also be advantageous in an agonistic context. In geese, serial agonistic interactions towards the same opponent are common [24] and attacks against the pair-partner of a former opponent also occur, although they are rare (B. Weiβ, unpubl. data). Therefore, communicating distress towards other individuals (e.g. pair-partners) may be important in order to prepare for action, namely to actively support the partner in an encounter. Thus, the increase in SDBs after stressful events might function to signal distress to other group members and therefore play an important communicative role.

In sum, our results show for the first time in non-human animals that the frequency and duration of certain post-conflict self-directed behaviors (shaking, autopreening) are correlated with autonomic arousal following an agonistic encounter and therefore may provide useful measures for estimating how an individual perceives a stressful event.

## References

[1] Wascher AFC, Scheiber IBR, Weiβ BM, Kotrschal K (2009). Heart rate responses to agonistic interactions in greylag geese (*Anser anser*).. Animal Behaviour.

[2] Wascher CAF, Scheiber IBR, Braun A, Kotrschal K (in press). Heart rate responses to experimentally induced challenge situations in greylag geese (*Anser anser*).. Journal of comparative psychology.

[3] Tinbergen N (1952). “Derived” activities; their causation, biological significance, origin, and emancipation during evolution.. The Quaterly Review of Biology.

[4] Maestripieri D, Shino G, Aureli F, Troisi A (1992). A modest proposal: displacement activities as an indicator of emotions in primates.. Animal Behaviour.

[5] Aureli F, Yates K (2010). Distress prevention by grooming others in crested black macaques.. Biology Letters.

[6] Castles DL, Whiten A, Aureli F (1999). Social anxiety, relationship and self-directed behaviour among wild female olive baboons.. Animal Behaviour.

[7] Kutsukake N (2006). The context and quality of social relationships affect vigilance behaviour in wild chimpanzees.. Ethology.

[8] Troisi A (2002). Displacement activities as a behavioural measure of stress in nonhuman primates and human subjects.. Stress: The international journal on the biology of stress.

[9] Kalueff AV, Tuohimaa P (2004). Grooming analysis algorithm for neurobehavioural stress research.. Brain Research Protocols.

[10] Nephew BC, Aaron RS, Romero LM (2005). Effects of arginine vasotocin (AVT) on the behavioral, cardiovascular, and corticosterone response of starlings (*Sturnus vulgaris*) to crowding.. Hormones and Behavior.

[11] Nephew BC, Romero LM (2003). Behavioral, physiological, and endocrine responses of starlings to acute increases in density.. Hormones and Behavior.

[12] Nephew BC, Kahn SA, Romero LM (2003). Heart rate and behavior are regulated independently of corticosterone following diverse acute stressors.. General and Comparative Endocrinolology.

[13] Schino G, Perretta G, Taglioni A, Monaco V, Troisi A (1996). Primate displacement activities as an ethopharmacological model of anxiety.. Anxiety.

[14] Riters LV, Panksepp J (1997). Effects of vasotocin on aggressive behavior in male Japanese quail.. Annals of the New York Academy of Sciences.

[15] Mason GJ (1991). Stereotypies: a critical review.. Animal Behaviour.

[16] de Waal FBM, van Roosmalen A (1979). Reconciliation and consolation among chimpanzees.. Behavioral Ecology and Sociobiology.

[17] Aureli F, Van Schaik CP (1991). Post-conflict behaviour in long-tailed macaques (Macaca fascicularis): II. Coping with the uncertainty.. Ethology.

[18] Castles DL, Whiten A (1998). Post-conflict behaviour of wild olive baboons. I. Reconciliation, redirection and consolation.. Ethology.

[19] Kutsukake N, Castles DL (2001). Reconciliation and variation in post-conflict stress in Japanese macaques (*Macaca fuscata fuscata*): testing the integrated hypothesis.. Animal Cognition.

[20] Fraser ON, Stahl D, Aureli F (2008). Stress reduction through consolation in chimpanzees.. Proceedings of the National Academy of Science.

[21] Butovskaya ML (2008). Reconciliation, dominance and cortisocl levels in children and adolescents (7-15-year-old boys).. Behaviour.

[22] Kotrschal K, Scheiber IBR, Hirschenhauser K, Kappeler P (2010). Individual performance in complex social systems.. Animal Behaviour: Evolution & Mechanism.

[23] Scheiber IBR, Kotrschal K, Weiβ BM (2009). Benefits of family reunions: social support in secondary greylag goose families.. Hormones and Behavior.

[24] Scheiber IBR, Kotrschal K, Weiβ BM (2009). Serial agonistic attacks by greylag goose families, *Anser anser*, against the same opponent.. Animal Behaviour.

[25] Scheiber IBR, Weiβ BM, Frigerio D, Kotrschal K (2005). Active and passive social support in families of greylag geese (*Anser anser*).. Behaviour.

[26] Weiβ BM, Kotrschal K, Frigerio D, Hemetsberger J, Scheiber IBR, Ramirez RN (2008). Birds of a feather stay together: extended family bonds, clan structures and social support in greylag geese (*Anser anser*).. Family Relations Issues and Challenges.

[27] Wascher AFC, Arnold W, Kotrschal K (2008). Heart rate modulation by social contexts in greylag geese (*Anser anser*).. Journal of Comparative Psychology.

[28] Hemetsberger J, Scheiber IBR, Weiβ BM, Frigerio D, Kotrschal K (2010). Influences of socially involved hand-raising on life history and stress responses in greylag geese.. Interaction studies.

[29] Weiβ BM, Kotrschal K (2004). Effects of passive social support in juvenile greylag geese *(Anser anser*): A study from fledging to adulthood.. Ethology.

[30] Scheiber IBR, Kralj S, Kotrschal K (2005). Sampling effort/frequency necessary to infer individual acute stress responses from fecal analysis in greylag geese (*Anser anser*).. Annals of the New York Academy of Sciences.

[31] Prinzinger R, Nagel B, Bahat O, Bögel R, Karl E (2002). Energy metabolism and body temperature in the griffon vulture (*Gyps fulvus*) with comparative data on the hooded vulture (*Necrosyrtes monachus*) and the white-backed vulture (*Gyps africanus*).. Journal of Ornithology.

[32] Noldus, In (2002). The observer ®.. 4.1 ed.

[33] de Waal FBM, Yoshihara D (1983). Reconciliation and redirected affection in rhesus monkeys.. Behaviour.

[34] Burnham KP, Anderson DR (2004). Multimodel inference: understanding AIC and BIC in model selection.. Sociological methods & research.

[35] Tabachnick BG, Fidell LS (2007). Using Multivariate Statistics..

[36] Major P (1998). Subtle physical activity poses a challenge to the study of heart rate.. Physiology & Behavior.

[37] Wascher AFC, Scheiber IBR, Kotrschal K (2008). Heart rate modulation in bystanding geese watching social and non-social events.. Proceedings of the Royal Society of London, Series B.

[38] Johnstone RA (2001). Eavesdropping and animal conflict.. Proceedings of the National Academy of Science.

[39] Peake T, McGregor PK (2005). Eavesdropping in communication networks. Animal communication networks..

[40] Preston SD, de Waal FBM (2002). Empathy: Its ultimate and proximate bases.. Behavioral and Brain Sciences.

[41] de Waal F (2008). Putting the altruism back into altruism: The evolution of empathy.. Annual Review of Psychology.

[42] Aureli F (1997). Post-conflict anxiety in nonhuman primates: The mediating role of emotion in conflict resolution.. Aggressive behaviour.

[43] de Gelder B, Snyder J, Greve D, Gerard G, Hadjikhani N (2004). Fear fosters flight: A mechanism for fear contagion when perceiving emotion expressed by a whole body.. Proceeding of the National Academy of Science.

[44] Parr LA, Wallner BM, Fugate J (2006). Emotional communication in primates: implications for neurobiology.. Current opinion in neurobiology.

[45] Spoor JR, Kelly JR (2004). The evolutionary significance of affect in groups: communication and group bonding.. Group Processes & Intergroup Relations.

[46] Tinbergen N (1963). On aims and methods of ethology.. Zeitschrift für Tierpsychologie.

